# Enhancing access to nephrology care: telenephrology dashboard optimization via human-centered design

**DOI:** 10.1186/s12882-025-04076-5

**Published:** 2025-03-31

**Authors:** Melissa Swee, Bharat Kumar, M. Lee Sanders, Angie Thumann, Kantima Phisitkul, Benjamin R. Griffin, Masaaki Yamada, Meenakshi Sambharia, Mary Vaughan-Sarrazin, Heather S. Reisinger, Bradley S. Dixon, Diana I. Jalal

**Affiliations:** 1 Division of Nephrology, University of Iowa Carver College of Medicine, 200 Hawkins Drive, Iowa City, IA 52245 USA; 2 Center for Access Delivery & Research and Evaluation (CADRE) Center, Iowa City VA Health Care System, Iowa City, IA USA; 3 Division of Immunology, University of Iowa Carver College of Medicine, Iowa City, IA USA; 4 Iowa City Veterans Affairs Health Care System, Iowa City, IA USA; 5 Institute for Clinical and Translational Science, University of Iowa, Iowa City, IA USA

**Keywords:** Telenephrology, Quality improvement, Dashboard, Chronic kidney disease, Clinical decision support, Telehealth

## Abstract

**Background:**

Ensuring that patients, especially those in underserved areas, have access to specialized nephrology care is essential to addressing the increasing burden of chronic kidney disease. To address this, we developed the Telenephrology Dashboard for the 150,000 Veterans served by the Iowa City Veterans Affairs Health Care System (ICVAHCS). Our goal was to optimize the dashboard as a comprehensive and practical tool for end-users in order to monitor kidney health and facilitate remote nephrology consultations.

**Methods:**

The optimization process adhered to the Human-Centered Design (HCD) framework, encompassing five stages: Empathize, Define, Ideate, Prototype and Test. Research team members spent 10 h observing nephrologists during remote consultations and supplemented these observations with semi-structured interviews with clinicians to gain insights into existing workflows and challenges. A rapid ideation workshop was then held to propose innovative solutions that balanced technical needs with user preferences. Subsequent prototyping and testing helped refine and evaluate the proposed designs, identifying key areas for improvement.

**Results:**

The iterative design process identified three critical needs: (1) improved clarity in visual data representation, (2) enhanced data accuracy, and (3) a balance between standardized features and customization options. Five dashboard prototypes were created, tested, and iteratively refined into a final version. The completed Telenephrology Dashboard includes five core features: (1) graphical representation of kidney function trends, (2) tables summarizing key lab data, (3) functionality to examine specific events in detail, (4) customizable views tailored to user workflows, and (5) integration of predictive kidney disease progression models.

**Conclusion:**

The Telenephrology Dashboard was developed using a Human-Centered Design approach to improve remote nephrology consultations. Future efforts will focus on evaluating its impact on user satisfaction, referring clinician satisfaction, access to nephrology care, and patient care outcomes.

**Clinical trial number:**

Not applicable, as this is not a clinical trial.

**Supplementary Information:**

The online version contains supplementary material available at 10.1186/s12882-025-04076-5.

## Introduction

Nephrologists play important roles in the prevention and management of acute and chronic kidney diseases and add considerable value to the multidisciplinary care of patients who have kidney disease [1, 2]. However, access to kidney specialists has been a major public health problem, especially for underserved rural areas [3]. The advent of telenephrology, a healthcare delivery model utilizing advanced telecommunications technology to provide nephrology care remotely, has emerged as a promising solution to these challenges [4]. This modality expands the reach of nephrology specialists and may enhance patient access to timely and expert care for frequent monitoring and complex management [5–7].

The COVID-19 pandemic accelerated the adoption of telehealth services across various medical fields, including nephrology [8]. The necessity of maintaining continuity of care while minimizing virus transmission, combined with advancements in telecommunications and investments into telecommunications infrastructure, have prompted a rapid integration of telehealth into everyday clinical practice [9]. This shift may be particularly beneficial in rural settings, where disparities in healthcare access have been most pronounced [10, 11].

Despite its growing adoption, several obstacles have hampered more widespread implementation of telenephrology [12]. Key among these is the lack of comprehensive tools to facilitate effective remote consultations. Many existing systems are not optimized for nephrology-specific needs, such as detailed monitoring of kidney function and comorbidities, which are vital for tracking the health of patients with kidney disease [13]. Additionally, there is a significant gap in research concerning the practical deployment of telenephrology interventions, with few studies rigorously addressing the effective integration of such systems into existing healthcare frameworks [14].

Recognizing these challenges, our project aimed to develop a Telenephrology Dashboard for Veterans receiving care through the Iowa City Veterans Affairs Health Care System (ICVAHCS). The dashboard was designed as an independent system, distinct from existing electronic health record (EHR) tools, to enhance early recognition and treatment of chronic kidney disease (CKD). Developed specifically for remote nephrology consultations, it addresses the unique challenges of managing progressive kidney disease in a virtual setting. By providing structured risk stratification and trend analysis, the dashboard enables primary care clinicians to identify high-risk patients earlier, facilitating timely interventions and improved coordination with nephrology specialists.

The initial creation followed Lean Six Sigma methodology, as our team previously described, to systematically identify and address workflow inefficiencies [13]. Building upon this foundational work, we employed a human-centered design (HCD) thinking process to refine the dashboard’s effectiveness and usability. This iterative improvement process was guided by rapid qualitative analysis techniques and the Consolidated Framework for Implementation Research (CFIR), ensuring that the final version met the specific demands of remote nephrology care [15, 16].

## Methods

This study was reviewed by the Institutional Review Board (IRB) of the Iowa City Veterans Affairs Health Care System (University of Iowa IRB-03 under the Human Subjects Office). As a quality improvement project, it was determined to be exempt from IRB review under the provisions of 45 CFR 46 Sect. 101(b). The need for informed consent was waived based on this determination. No direct patient observation or interaction occurred; only clinician workflows were evaluated, and no patient data was collected.

### Settings and participants

The ICVAHCS is a member of the Veterans Affairs Midwest Health Care Network serving over 184,000 Veterans throughout Eastern Iowa, Western Illinois, and Northern Missouri. The population is predominantly rural. The hospital and subspecialty clinics, such as nephrology, are based in Iowa City, with 13 community-based outpatient clinics (CBOCs) in Iowa and Illinois that deliver primary care [17]. When patients require specialist care, they are referred to the Iowa City clinics or receive treatment through community providers outside the VA system.

### Study design

The Telenephrology Dashboard was developed independently of any existing EHR system. It was initially created from the ground up using Lean Six Sigma methodology, which we previously published, to systematically identify workflow challenges and optimize processes [13]. The dashboard was further refined using Human-Centered Design principles to ensure usability for nephrologists and primary care clinicians, particularly in telenephrology settings.

Lab data is securely extracted from the VA Corporate Data Warehouse, which is the source of laboratory data in the EHR, ensuring compliance with VA data protection standards. Data transmission adheres to HIPAA regulations, with access restricted to credentialed nephrology providers. This integration allows the Telenephrology Dashboard to synthesize and present relevant clinical data efficiently while maintaining strict security and privacy standards.

**Fig. 1:**
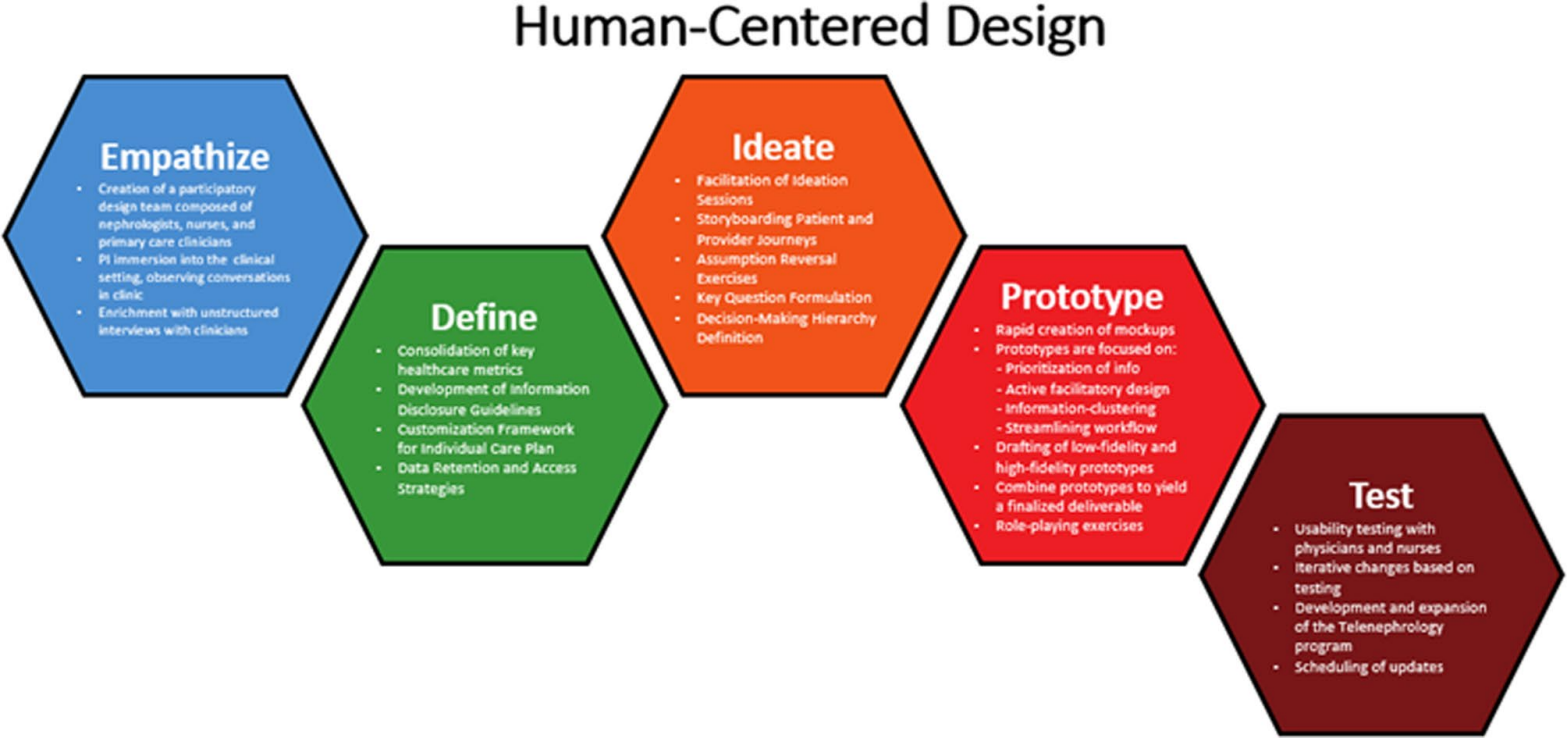
Human-centered design process for telenephrology dashboard development. The design team employed an iterative and nonlinear process developed by the Hasso Plattner Institute of Design at Stanford (d.School). The five stages of this design thinking process included Empathize, Define, Ideate, Prototype, and Test

Human-Centered Design is an approach towards problem-solving that develops solutions to problems by prioritizing human perspectives throughout all elements of the design process [18]. Design thinking depends upon the cultivation of deep empathy for users to empower a rigorous co-design process involving both participants and designers [19]. The approach is heterogeneous and there are several frameworks and models for the application of human-centered design principles [20]. In this project, we utilized design thinking model proposed by the Hasso Plattner Institute of Design at Stanford (d.school) consisting of five stages: Empathize, Define, Ideate, Prototype, and Test (Fig. 1) [21].

### Empathize

The Empathize stage was aimed at understanding the experiences, challenges, and needs of health care team members who conduct remote nephrology consultations. The design team engaged in active observation as nephrologists, case managers, and primary care clinicians engaged in the process of obtaining data, synthesizing information, generating recommendations, and relaying the management plan. Observers were instructed to take field notes and tie them to specific instances and observations.

To augment these observations, design team members also conducted semi-structured interviews with five nephrologists and ten primary care clinicians, focusing on how clinicians interact with the health care system to choose a treatment option for kidney disease. The interview guides for these semi-structured interviews are available in the supplementary material. To structure the analysis of these interviews, Hamilton’s Rapid Qualitative Analysis (RQA) was utilized [22]. Rapid qualitative analysis is comparable to traditional qualitative methods for the purposes of quality improvement and implementation [23]. 

To operationalize RQA within the Empathize stage of this design thinking process, we approached the process of interview analysis in three steps. First, neutral domain names were created corresponding with each interview question. Then, the design team developed an interview summary template. This template was applied and revised by the team for usability, relevance, readability, organization, and thoroughness. Finally, for each interview, summary points were transferred to a matrix for rapid qualitative analysis.

### Define

In the Define stage, the design team synthesized the information gathered during the Empathize phase to clearly outline the core issues affecting shared decision-making in the clinic. Using the empathy map and examining other field notes, the design team crystallized an initial set of human-centered problem statements. To further refine these problem statements, the QI team used “how might we” questions and “why-how laddering” until achieving consensus about the future direction of the project [24].

### Ideate

During the Ideate stage, the design team generated a range of potential solutions to address the defined set of problems. A series of rapid ideation workshops sessions were convened that included an interprofessional and multidisciplinary group of individuals to ensure a wide array of perspectives and ideas. The creative brainstorming process aimed to produce innovative yet feasible solutions. Ideation was facilitated by several activities, including analogizing, SCAMPER (Substitute, Combine, Adapt, Modify, Put to another Use, Eliminate, and Reverse), and brainwalking [25]. Throughout the Ideate stage, the principles of convergent and divergent thinking were employed to iteratively redefine the focus and scope for the Prototype stage.

### Prototype

The Prototype stage involved the elaboration of selected ideas from the Ideate phase to develop several low-fidelity prototypes. Using information from the rapid ideation sessions, the design team created mockups of the emerging Telenephrology Dashboard. These prototypes focused on five elements: (1) graphical display of kidney function over time, (2) tables synthesizing lab data, (3) options to drill down events to specific times, (4) customization of views, and (5) integration of kidney disease progression models. Low-fidelity and high-fidelity mockups were drafted and evaluated to critically appraise how these mockups would help answer the problem statements in the Define stage and impact the experience of conducting a telenephrology consultation as noted in the Empathize stage. Principles of the SCAMPER method were cross-applied here in order to iterate more refined versions.

### Test

In the Test stage, the design team conducted comprehensive evaluations of refined prototypes with a larger group of participants in the clinic setting. Testing assessed the usability, clarity, and overall impact of the high-fidelity prototypes on the ability to conduct telenephrology consultations. Feedback was collected through informal feedback and observations during follow-up discussions. Because the design thinking process is not meant to be sequential and linear, the Test stage led to revisiting elements of the Empathize and Define stages.

## Results

### Participants

This quality improvement project involved multiple key stakeholders within ICVAHCS. Initially, the Telenephrology Dashboard was implemented within four CBOCs as part of the pilot phase as previously described in a separate manuscript [13]. Following successful implementation and iterative improvements, the Telenephrology Dashboard was expanded to include all 13 CBOCs across the Iowa City VA system.

Primary care practitioners (PCPs) and nephrologists were involved throughout the process. In total, 61 primary care practitioners and 5 nephrologists, serving over 50,000 patients, participated in these quality improvement efforts.

### Human centered design

#### Empathize

**Fig. 2:**
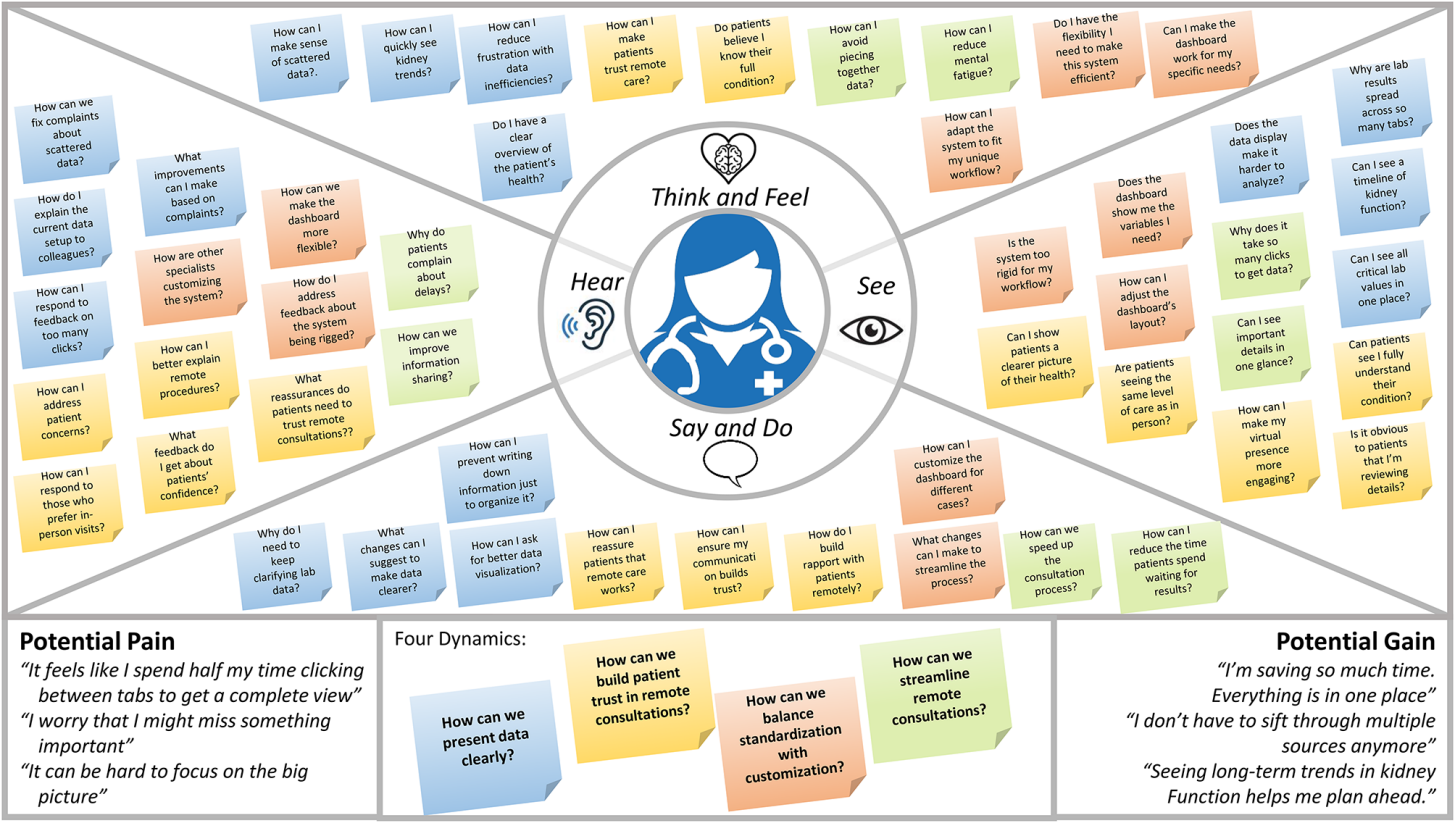
Clinician Empathy Map. The clinician empathy map captures key insights into nephrologists’ thoughts, visual interactions, feedback, and actions while using the Telenephrology Dashboard. It is organized across four dynamics: presenting data clearly, building patient trust in remote care, balancing standardization with customization, and streamlining consultations. These categories help identify challenges and opportunities to improve the provider experience and enhance the dashboard’s effectiveness

During the Empathize stage, two members of the design team (BK and MS) engaged in active observation of 20 telenephrology consultations. This totaled approximately 10 h of direct observation. In addition, there were 15 semi-structured interviews with clinicians to debrief the decision-making process. Based on these results, an empathy map was constructed (Fig. 2), showing what clinicians see, think, do, and feel when interacting with the health care system. The map highlights a significant emotional and cognitive load on clinicians, and a need for clinicians for user-friendly tools that facilitate synthesis of information and guide decision support.

#### Define

Through analysis of the empathy maps and other observational data, the design team crystallized three problem statements: (1) improved clarity in visual data representation, (2) enhanced data accuracy, and (3) a balance between standardized features and customization options.

#### Clarity in visualizing data

While observing nephrologists during their telenephrology consultations, the design team noticed inefficiencies and complexities in gathering data necessary for a consultation. Although critical information was available in the electronic health record system, it was scattered across different sections, requiring numerous clicks to access and compile. To make sense of this data, nephrologists often manually noted key values along with dates and times, sketching ad hoc charts to help visualize trends for analysis. The use of arrows and annotations underscored the non-linear, intricate nature of this workflow, which demanded data integration and spatial visualization rather than simple copying.

This was corroborated in the semi-structured interviews, in which nephrologists had mentioned that there was a lot of sophistication in generating these summaries of information and interrogating the information in the summary with newer information. There was also concern that the drafts were not truly “twenty-first century” and “there has to be a better way.”

#### Accuracy of information

Nephrologists also mentioned that there was concern about the accuracy of the information provided. Since their consultations rely heavily on the integrity of the data received, they emphasized the critical need for reliable data. While they generally trusted the information within the electronic health record, semi-structured interviews revealed some apprehension about how certain data representations might affect accuracy. One nephrologist illustrated this by using an example in the electronic health record: “Look at the axis here… it doesn’t start at 0, so it looks like this is a gigantic jump in creatinine… but it’s more modest when you actually look at the numbers.”

#### Balancing standardization with individualization

Design team members also noted that there was heterogeneity in the way that nephrologists approached and synthesized the data. Or as one participant stated: “There is no one way to do things correctly here but there are many ways to do things incorrectly.” In observing telenephrology consultations, design team members compared and contrasted the approaches that nephrologist undertook, anticipating how workflow would be accommodated in a future prototype. From these observations and results of semi-structured interviews, it became clear that the tool had to be multifaceted in accommodating many different ways of looking at data and supporting clinicians in reliably synthesizing that information.

#### Ideate

To generate potential solutions and pathways forward to these four problem statements, the design team convened a series of three rapid ideation workshops. During these sessions, design team members engaged in creative brainstorming using three major tools: (1) Analogizing, (2) Brainwalking, and (3) SCAMPER.

#### Analogizing

Analogizing helped to view telenephrology consultations through different lenses. Three lenses were adopted to help understand the mindset of nephrologists during consultations: (1) as a conventional face-to-face visit, (2) as a detective story, and (3) as a video game. These analogies helped to highlight different dynamics and expectations during consultations, although design team members were careful to note that few consultations fit into strictly one category.

#### Brainwalking

To promote unconventional thinking and creativity, the design team engaged in a brainwalking session. The seven rules of brainwalking were strictly adhered to: (1) defer judgment, (2) go for volume, (3) one conversation at a time, (4) encourage wild ideas, (5) build on the ideas of others, (6) stay on topic, and (7) be visual. The problems noted in the Define phase were reformulated in the “how might we…” context, i.e. how might we: (1) clearly visualize relevant data, (2) accurately display and collate representations of information, and (3) balance standardization of the tool with individualized workflows and patterns? Flipcharts for each of these three statements were silently filled, with only one minute allotted per flip chart. Once completed, the drawings, which included very preliminary prototypes, were collected and reconciled.

#### SCAMPER

Because this was likely to be a digital product, the design team utilized the SCAMPER method to arrive at fresh insights through a structured approach to ideation [26]. Using the early emerging prototypes from the brainwalking session and the electronic health record displays, the design team first looked at ways to SCAMPER certain features. For example, the design team saw that nephrologists were often looking at several datasets united by a single timeline. They deduced that a product that combines these many datasets in a visually appealing manner centered around a single timeline would be most useful. Similarly, the design team considered the modification of the existing graphs so that they would be more intuitive and graphically consistent among different views.

#### Prototype

**Fig. 3:**
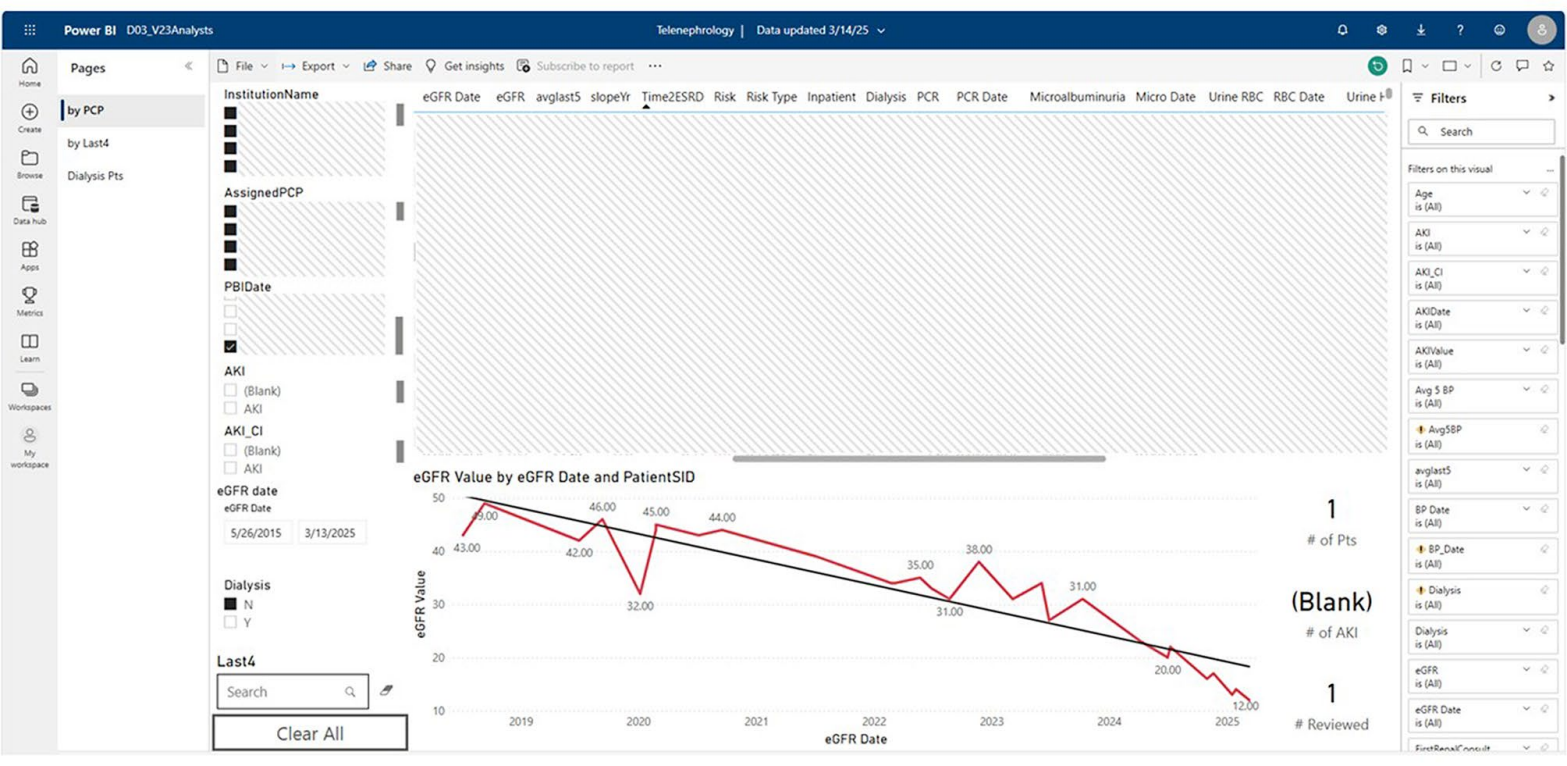
Finalized Prototype of the Optimized Telenephrology Dashboard. The finalized prototype included five features that were generated in the Ideate stage: (1) Graphical display of kidney function, (2) Tables synthesizing lab data, (3) Options to drill down events to specific times, (4) Customization of views, and (5) Integration of kidney disease progression models

The design team used insights generated from the Ideate stage to develop 5 prototypes, which were reconciled to yield the finalized prototype (Fig. 3).

#### Graphical display of kidney function

The graphical display of kidney function over time was designed to visually capture trends in estimated glomerular filtration rate (eGFR). This feature allowed nephrologists to quickly identify significant changes or patterns in kidney function and correlated them with other clinical variables. Nephrologists appreciated the ability to see kidney function data in a clear, easy-to-interpret format, which helped guide their clinical decision-making during consultations.

#### Tables synthesizing lab data

The tables synthesizing lab data were developed to present essential laboratory results in a concise, organized manner. These tables provided a snapshot of key values such as blood pressure, presence of hematuria, and last evaluation of microalbuminuria. By consolidating lab results into one view, nephrologists could easily compare relevant data without needing to navigate through multiple screens or sources.

#### Options to drill down events to specific times

The option to drill down into specific events allowed nephrologists to explore data in more granular detail when needed. By selecting particular dates or time frames, nephrologists could analyze lab results and changes in patient status. This feature enhanced their ability to contextualize kidney function trends with real-world events.

#### Customization of views

Customization of views empowered nephrologists to tailor the dashboard to their specific preferences and workflows. Users could adjust the layout, select which data points to emphasize, and create personalized templates for different types of consultations. This flexibility was particularly valued as it allowed clinicians to optimize the dashboard for their individual practice styles while maintaining consistency in data presentation.

#### Integration of kidney disease progression models

The integration of kidney disease progression models within the dashboard provided predictive insights that could inform long-term care planning. By incorporating established algorithms, the dashboard enabled nephrologists to assess future risks and outcomes for patients based on current data [27]. This feature helped facilitate discussions around prognosis and treatment decisions with patients and their care teams, promoting a more proactive approach to kidney care management.

### Test

Once the optimized version of the Telenephrology Dashboard was created, the design team conducted usability testing with 5 individuals. The dashboard was modified slightly with additional feedback, especially to enable greater customization of views, as noted in the third human-centered problem statement.

Observations of nephrologists utilizing the Telenephrology Dashboard demonstrated that once oriented to its features, nephrologists were better empowered to synthesize multiple sources of information. The consolidation of information especially enabled nephrologists to analyze trends in kidney function with respect to other changes in the health status of Veterans. Further interviews suggested that nephrologists themselves felt a greater sense of flow in their work: “There’s less interruptions for sure so I can be the nephrologist I want to be, and that means that patients get better care.”

An unanticipated benefit of the Telenephrology Dashboard has been a greater engagement with the data by the nephrologists. By clearly visualizing the data, particularly correlating temporal events to laboratory results, it has heightened awareness of certain connections that led to feedback loops for continuing self-education. One concern that was mentioned was over-reliance on the dashboard, as there may be other sources of information that are not entered into the Telenephrology Dashboard which are important and may be missed.

## Discussion

In this manuscript, we report the optimization of the Telenephrology Dashboard, which represents an advancement in the field of telenephrology. By harmonizing software development with human systems engineering, we have created a tool tailored to the unique demands of remote nephrology care. Our human-centric design thinking approach has addressed both technical and practical challenges, providing new insights into how telenephrology solutions can be integrated into routine clinical practice. This novel approach not only improves the functionality of telehealth tools but also enhances the capacity for clinicians to engage in nephrology care.

Data show that the involvement of kidney specialists helps to reduce progression to end-stage kidney disease [28]. However, due to the current workforce shortage and geographic disparities, patients who are vulnerable to progression of their kidney disease are unlikely to have access to specialists who can engage in continual and detailed monitoring [29]. 

The Telenephrology Dashboard can be part of a solution to this problem by enabling telenephrology consultations to be more efficient and more targeted to healthcare issues [5]. Unlike standard EHR systems that require nephrologists to navigate multiple sections and manually compile data for trend analysis, the dashboard integrates relevant kidney function parameters into an intuitive single-screen display. This consolidation aids in reducing the cognitive load on clinicians, allowing for more focused and informed decision-making processes, as evidenced by the reception of the dashboard’s graphical displays and integrated data views among nephrologists.

One of the insights of this project is how the implementation of relatively simple human-centric design thinking principles can lead to tools that enhance clinical decision-making efficiency. The dashboard eliminates the need for manual compilation of lab values and facilitates rapid trend recognition by incorporating graphical representations of eGFR and serum creatinine fluctuations. This feature reduces time spent retrieving and interpreting data, ultimately enabling clinicians to provide more timely and informed recommendations to referring providers. In addition to improving the quality of patient care, this efficiency may also improve job satisfaction. Clinician satisfaction with such dashboards has been observed in other healthcare settings, where usability and engagement with these tools have been linked to increased efficiency, reduced cognitive load, and improved decision-making accuracy​. Notably, engaging providers in the design process, as we did in this project, has been highlighted as a major factor for successful dashboard implementation and adoption [30, 31]. 

Currently, the dashboard is clinician-facing, designed to optimize nephrology workflows. Future iterations may explore patient access features to enhance patient engagement and shared decision-making in CKD management.

Despite the benefits that were observed during the Test stage, several barriers to the widespread adoption of such technology persist. These include the potential for increased reliance on one single technological solution and the sustainability of telehealth programs in terms of funding and resource allocation. Moreover, implementation of telehealth solutions without careful evaluation of user engagement and system usability can compromise the success of these technologies. Evidence from similar dashboard implementations indicates that rushed deployments, without involving end-users in iterative design processes, can lead to poor adoption and negative outcomes [30, 31]. Therefore, it is crucial that healthcare systems adopt a deliberate and collaborative approach, ensuring that continuous feedback loops between end-users and dashboard developers are maintained to keep the tool responsive to evolving needs​.

The future of telenephrology, particularly with tools like the Telenephrology Dashboard, appears promising yet demands concerted efforts for broader implementation and continuous improvement. Future research should focus on long-term outcomes of using such telehealth solutions, including their impact on patient health outcomes, satisfaction levels, and overall healthcare system efficiency. Additionally, studies could explore the scalability of the dashboard to other medical specialties and settings, potentially broadening the scope of telehealth to benefit a wider array of patient populations and healthcare systems.

### Limitations

This quality improvement project, while demonstrating promising preliminary results, is not without limitations. First, this was a quality improvement project conducted within a single Veterans Affairs catchment area, which necessarily limits the generalizability of the findings. The patient population within this catchment area is unique in terms of demographics and healthcare access, which influences the applicability of the tools in other settings with different patient populations or in private practice environments.

Secondly, while the Telenephrology Dashboard has been designed to be user-friendly and intuitive, individual variations in technology acceptance and digital literacy among health care team members could affect the adoption rate and overall effectiveness of the tool. Resistance to adopting new technologies, due to either comfort with existing practices or skepticism towards new tools, could limit the utilization of the dashboard.

Finally, this project primarily focuses on the optimization of the dashboard, with less emphasis on measuring direct clinical outcomes. Future studies are needed to assess the impact of the dashboard on patient health outcomes, quality of care, and cost-effectiveness to fully understand its benefits and potential drawbacks.

## Conclusion

In conclusion, the Telenephrology Dashboard has demonstrated significant potential in enhancing nephrology care through improved data visualization, accuracy, and the facilitation of remote consultations. By addressing both technical and operational challenges, this project contributes to a more effective, efficient, and patient-centered healthcare delivery model. The continued evolution of this tool will undoubtedly hinge on its ability to adapt to the changing landscapes of healthcare needs and technological advancements, underscoring the importance of sustained innovation and research in telehealth.

## Electronic supplementary material

Below is the link to the electronic supplementary material.

Supplementary Material 1

Supplementary Material 2

## Data Availability

No datasets were generated or analysed during the current study.

## Abbreviations

ICVAHCS: Iowa City Veterans Affairs Health Care System
HCD: Human-Centered Design
IRB: Institutional Review Board
CBOCs: Community-Based Outpatient Clinics
CFIR: Consolidated Framework for Implementation Research
VA: Veterans Affairs
eGFR: Estimated Glomerular Filtration Rate
PCPs: Primary Care Practitioners
RQA: Rapid Qualitative Analysis
