# Supplementary material for: Enhancing access to nephrology care: telenephrology dashboard optimization via human-centered design
Source: BMC Nephrol. 2025 Mar 31;26:164. doi: 10.1186/s12882-025-04076-5 (PMC11956470; doi:10.1186/s12882-025-04076-5)
Supplement: Supplementary file 1 — Supplementary Material 1 [file 12882_2025_4076_MOESM1_ESM.docx]

**Interview Guide for Telenephrology Dashboard Evaluation for Nephrologists**

Thank you for participating in this interview. We are conducting this study to better understand how clinicians interact with the healthcare system and make decisions regarding kidney disease management. Your insights will help refine and optimize the Telenephrology Dashboard to better meet clinical needs.

This interview will take approximately 30 minutes. Your responses will be confidential, and no identifying information will be shared. We will be asking questions about your workflow, decision-making processes, and experiences using the Telenephrology Dashboard.

**Section 1: Background and Workflow**

1. Can you describe your typical workflow when conducting a nephrology consultation, either in person or via telenephrology?

2. What are the main challenges you face when synthesizing patient data for decision-making in nephrology?

3. How do you typically gather and organize relevant clinical information from the electronic health record (EHR)?

4. Have you previously used decision-support tools or dashboards in your clinical practice? If so, what aspects were most useful?

**Section 2: Experience with Data Visualization and Accuracy**

5. When reviewing lab data and trends in kidney function, what factors influence your decision-making process?

6. How do you currently visualize kidney function trends (e.g., changes in creatinine, estimated GFR, proteinuria)?

7. Are there specific aspects of data representation that make it easier or harder for you to interpret trends?

8. Have you encountered issues with data accuracy in the EHR or other clinical tools? If so, how did you address them?

9. Do you find that data representations (such as graphs or tables) sometimes lead to misinterpretation of trends? Can you provide an example?

**Section 3: Interaction with the Telenephrology Dashboard**

10. Have you used the Telenephrology Dashboard in your practice? If so, what was your initial impression?

11. How does the dashboard compare to your previous method of synthesizing patient information?

12. Which features of the dashboard have been most helpful for your workflow? Why?

13. Are there any features that you found confusing or difficult to use?

14. What aspects of the dashboard do you think should be improved or added?

15. How well does the dashboard integrate with your decision-making process during telenephrology consultations?

**Section 4: Standardization vs. Customization**

16. Do you think the dashboard provides the right balance between standardized data presentation and customizable features?

17. Are there specific ways you would like to customize the dashboard to better fit your needs?

18. How do you feel about the flexibility of the dashboard in accommodating different clinical workflows?

**Section 5: Impact on Clinical Decision-Making**

19. Has using the dashboard changed the way you approach nephrology consultations? If so, in what ways?

20. Has the dashboard helped reduce cognitive load or improved efficiency in consultations?

21. Have you noticed any improvements in communication with other clinicians due to the dashboard?

22. Have you identified any new patterns or insights in patient data because of the dashboard?

23. Do you foresee any potential downsides or risks of relying on the dashboard for decision-making?

**Section 6: Future Directions and Additional Feedback**

24. What additional features would you like to see in future iterations of the Telenephrology Dashboard?

25. Are there any specific challenges in telenephrology that the dashboard does not currently address?

26. What suggestions do you have for improving the usability and effectiveness of the dashboard?

27. Is there anything else you would like to share about your experience with the dashboard or nephrology consultations in general?

**Closing:**

Thank you for your time and valuable insights. Your feedback will help shape the development of the Telenephrology Dashboard. If you have any further thoughts or suggestions, please feel free to reach out to the telenephrology team.
