# Supplementary material for: Enhancing access to nephrology care: telenephrology dashboard optimization via human-centered design
Source: BMC Nephrol. 2025 Mar 31;26:164. doi: 10.1186/s12882-025-04076-5 (PMC11956470; doi:10.1186/s12882-025-04076-5)
Supplement: Supplementary file 2 — Supplementary Material 2 [file 12882_2025_4076_MOESM2_ESM.docx]

**Interview Guide for Primary Care Clinicians**

Thank you for participating in this interview. We are conducting this study to understand how primary care clinicians manage kidney disease in their patients and how telenephrology consultations impact care. Your insights will help refine our workflow and ensure that nephrology consults provide the most useful information to primary care teams. This interview will take approximately 15-20 minutes. Your responses will be confidential, and no identifying information will be shared.

**Section 1: Background and Workflow**

1. Can you describe your typical workflow when managing patients with kidney disease?

2. What are the main challenges you face when coordinating care for patients with chronic kidney disease (CKD)?

3. How do you typically receive nephrology recommendations, and how do you incorporate them into patient care?

**Section 2: Communication and Decision-Making**

4. What type of information is most helpful for you when managing a patient referred to nephrology?

5. Are there specific pieces of data or recommendations that are particularly useful or difficult to interpret?

6. Have there been any challenges in understanding or applying nephrology recommendations in patient care?

**Section 3: Reporting and Documentation**

7. What would you like to see in nephrology consultation notes to make them more useful for primary care clinicians?

8. Are there any gaps in the current documentation that make it harder to integrate recommendations into patient care plans?

**Section 4: Future Directions and Additional Feedback**

9. What improvements could be made to the nephrology consultation process to better support primary care clinicians?

10. Is there anything else you’d like to share about your experience with nephrology consultations or managing CKD patients?

**Closing:**

Thank you for your time and valuable insights. Your feedback will help us refine our communication and improve the quality of nephrology care coordination. If you have any further thoughts or suggestions, please feel free to reach out to the telenephrology team.
